# Assessing α-Bisabolol as a Transmucosal Permeation Enhancer of Buccal Local Anesthetics

**DOI:** 10.3390/pharmaceutics16091198

**Published:** 2024-09-12

**Authors:** Renê Oliveira do Couto, Douglas Vieira Thomaz, Maira Perez Ferreira Duarte, Renata Fonseca Vianna Lopez, Vinícius Pedrazzi, Osvaldo de Freitas, Gianluca Martino Tartaglia

**Affiliations:** 1 “Dona Lindu” Midwest Campus, Universidade Federal de São João del-Rei (UFSJ), Divinopolis 35501-296, MG, Brazil; 2 Department of Biomedical, Surgical and Dental Sciences, School of Dentistry, University of Milan, 20129 Milan, Italy; douglasvthomaz@gmail.com (D.V.T.); gianluca.tartaglia@unimi.it (G.M.T.); 3 School of Pharmaceutical Sciences of Ribeirao Preto, Department of Pharmaceutical Sciences, Universidade de São Paulo (USP), Ribeirão Preto 14040-900, SP, Brazil; maira@fcfrp.usp.br (M.P.F.D.); rvianna@fcfrp.usp.br (R.F.V.L.); ofreitas@fcfrp.usp.br (O.d.F.); 4 School of Dentistry of Ribeirao Preto, Department of Dental Materials and Prosthodontics, Universidade de São Paulo (USP), Ribeirão Preto 14040-904, SP, Brazil; pedrazzi@forp.usp.br; 5 UOC Maxillo-Facial Surgery and Dentistry Fondazione IRCCS Ca’ Granda Ospedale Maggiore Policlinico, 20122 Milan, Italy

**Keywords:** buccal drug administration, hypromellose derivatives, lidocaine, prilocaine drug combination, local anesthesia, volatile oil

## Abstract

Needle-free buccal anesthesia improves dental treatment outcomes for both patients and dentists. In this study, we report on an assessment of the enhancement effects of α-bisabolol on the in vitro transmucosal permeation of prilocaine hydrochloride (PCl) and lidocaine hydrochloride (LCl) from needleless buccal films. We also evaluated the mechanical properties of the film, which consisted of Methocel™ K100 LV as the film-forming polymer (3% m·m^−1^), PEG 400 as a cosolvent (15% m·m^−1^ based on drug loading), α-bisabolol (15 and 30% m·m^−1^ based on drug loading), and the drugs combined at a 1:1 ratio (15 mg·unit^−1^). The porcine esophageal epithelium was used as a membrane barrier, and artificial saliva was the release medium. After a 1 h experiment at 25 ± 2 °C, α-bisabolol significantly decreased, rather than enhanced, the permeation fluxes (five-fold), permeability coefficients (seven-fold), and retentions (two-fold) of both PCl and LCl through the epithelium, regardless of the concentration. Moreover, the resistance and flexibility of the films markedly decreased compared to those without α-bisabolol. Therefore, under the experimental conditions, using α-bisabolol as a buccal permeation enhancer for the hydrophilic local anesthetics PCl and LCl from buccal films is not feasible.

## 1. Introduction

The inability to achieve adequate pain control, as well as the possibility of contamination and poisoning due to occupational accidents during professional practice, remains a significant concern for dentists. The reasons why injections cause pain is the pH of the anesthetic solution and, above all, the force of the needle puncture on the oral mucosa and the pressure of the solution entering the area to be anesthetized. Therefore, the anesthetic technique used by the professional plays a fundamental role in this scenario [1,2].

Numerous studies have examined the effective use, design, and clinical benefits of innovative devices and techniques for oral anesthesia [2,3,4,5]. However, these advances have not yet become popular in dental practices, likely because of their high cost; therefore, dentists have been seeking affordable alternatives to minimize patient discomfort.

Developing mucoadhesive thin films as a needle-free strategy for administering local anesthetics in dentistry presents an innovative approach with several potential benefits in clinical practice. Such a drug delivery system circumvents various issues related to injectable anesthesia, enhancing patient safety, comfort, and compliance [6]. Many patients worldwide experience anxiety and fear associated with needle punctures [7]; as a non-invasive method, mucoadhesive films can significantly improve their quality of life. Mucoadhesive films can deliver anesthetics directly to the mucosal tissues, ensuring a targeted and efficient anesthetic effect. These films can be designed for rapid release, improved drug permeation, and retention in the epithelium, providing a swift onset and lasting for a desired duration [8,9].

These advantages are not limited to patients and may benefit dentists worldwide. Films are easy to handle and require no special skills for administration, unlike injections. Since eliminating needles reduces the risk of infection and other issues related to labor accidents following needle use [10], mucoadhesive films can establish a starting point for safer dental procedures.

Nonetheless, it is noteworthy to mention that they can cause allergic reactions due to the hypersensitivity of some patients to either the active pharmaceutical ingredient or excipients (such as polymers, plasticizers, flavorings, sweeteners, colorants, and preservatives) [11,12,13]. Moreover, during buccal film administration, patients may experience discomfort due to mechanical irritation. To circumvent these issues, mucoadhesive buccal films must be rationally designed to enable easy detachment from the placement site [14,15]. 

Since its approval by the FDA (Food and Drug Administration, Silver Spring, MD, USA) in 1996, the Dentipatch™ has been considered the “state of the art” in the field of topical anesthesia in the oral cavity [16]. It is a transmucosal patch composed of a mucoadhesive hydrophilic polymer (gum tragacanth), lidocaine (46.1 mg per 2 cm^2^ patch), lecithin, propylene glycol, glycerol, aspartame, and flavoring. This pharmaceutical technology has shown greater pre-anesthetic efficacy and acceptance in pediatric patients compared to some conventional formulations available on the market [17,18,19,20,21,22].

The EMLA^®^ cream and ORAQIX^®^ polymeric gel, both combining prilocaine and lidocaine (base form) at a concentration of 2.5% (5% total anesthetic), are also FDA-approved products marketed worldwide [23,24]. These aminoamide drugs are widely used for infiltrative local anesthesia in routine dental procedures and surgical interventions due to their rapid onset (2–5 min) and medium duration of the anesthetic effect [25,26].

Given the balance of biopharmaceutical and pharmacological properties, combining these drugs provides enhanced anesthetic effects compared to their separate use [9,27]. Lidocaine is more lipophilic (having a greater partition coefficient [logP] and distribution coefficient [logD] at physiological pH) and, therefore, has greater potency, while prilocaine has a slower onset due to its higher pKa value. On the other hand, prilocaine has half the vasodilatory effect of lidocaine, which can prolong the anesthetic effect [28,29]. 

To the best of our knowledge, neither Dentipatch^TM^ nor EMLA^®^ or ORAQIX^®^ has effectively replaced infiltrative anesthesia by promoting nerve block. This leaves room for formulation scientists in academia and industry to investigate strategies for enhancing the permeation and retention of these local anesthetics across the buccal epithelium. In this context, various methods can be used to overcome the barrier imposed by the epithelium of the oral mucosa, including chemical permeation enhancers [30,31], microneedles [32,33,34,35,36], iontophoresis [37,38,39], nanoparticles [40,41,42,43,44,45,46], and mainly mucoadhesive delivery systems [47,48,49,50,51,52,53]. Combining some of these strategies has also been considered [30,31,54,55]. 

Alpha-bisabolol (α-bisabolol), a sesquiterpene found in the essential oils of chamomile (*Matricaria recutita*; Asteraceae) and rose (*Rosa damascena*; Rosaceae) [56], has shown promising potential as a chemical permeation enhancer for hydrophilic drug delivery through the skin [57] and buccal mucosa [58]. In the former, pretreatment with α-bisabolol significantly increased the logP, permeation flux, diffusion coefficient, and permeation coefficient of propranolol hydrochloride (PHCl). In the latter, this increased the retention of 5-aminolevulinic acid (5-ALA) in the epithelium by six-fold. These enhancements were likely achieved by modifying the solvent nature, an outstanding mechanism by which terpenes act [59]. Being a natural compound, α-bisabolol is generally considered safe and well-tolerated [60], making it advantageous over synthetic permeation enhancers that may irritate [61]. 

Drug permeation through buccal mucosa differs from skin permeation, since these physiological barriers present distinct biochemical compositions and architectures [62]. Therefore, the enhancement efficacy of a chemical can be specific to both the drug and administration site. Accordingly, depending on the drug delivery route, the same enhancer can have a different efficacy for a particular permeant [63]. Given this, is using α-bisabolol as a buccal permeation enhancer of local anesthetics worthwhile? This issue remains unexplored. 

Here, we investigated the in vitro efficacy (or lack thereof) of α-bisabolol as a permeation and retention enhancer of prilocaine hydrochloride (PCl) and lidocaine hydrochloride (LCl) through the buccal epithelium. Moreover, we examine the effects of increasing concentrations of this phytochemical on the mechanical properties of needle-free mucoadhesive films.

## 2. Materials and Methods

### 2.1. Chemicals and Drugs

Methocel™ K100 LV Premium (lot WP437291) was a gift from Colorcon^®^ (Cotia, SP, Brazil). Lidocaine hydrochloride (LCl) and prilocaine hydrochloride (PCl) were obtained from Henrifarma^®^ (São Paulo, SP, Brazil). High-performance liquid chromatography (HPLC)-grade methanol and acetonitrile were purchased from J.T. Baker^®^ (Phillipsburg, NJ, USA). Polyethylene glycol 400 (PEG 400), sodium phosphate dibasic (Na_2_HPO_4_), potassium phosphate monobasic (KH_2_PO_4_), sodium chloride (NaCl), and magnesium nitrate hexahydrate (Mg(NO_3_)_2_·6H_2_O) were sourced from Merck (Merck KGaA, Darmstadt, Germany). Analytical standards of PCl, LCl, α-bisabolol (purity > 93%), and diethylamine were obtained from Sigma-Aldrich (St. Louis, MI, USA). Ultrapure water was obtained by a Milli-Q^®^ system (Millipore, Bedford, MA, USA).

### 2.2. Manufacture and Quality Assessment of Buccal Anesthetic Films

The buccal anesthetic films were prepared by solvent casting [9]. Table 1 displays the composition of the different films in development. Briefly, LCl and PCl were dissolved in a 0.1 M sodium phosphate buffer (pH 7.0) containing the cosolvent (PEG 400). Then, the permeation enhancer α-bisabolol was added dropwise to this solution under stirring (500 rpm). Next, the film-forming hydrophilic polymer was dispersed, and the system was maintained under mechanical stirring (300 rpm) overnight.

**Figure 1:**
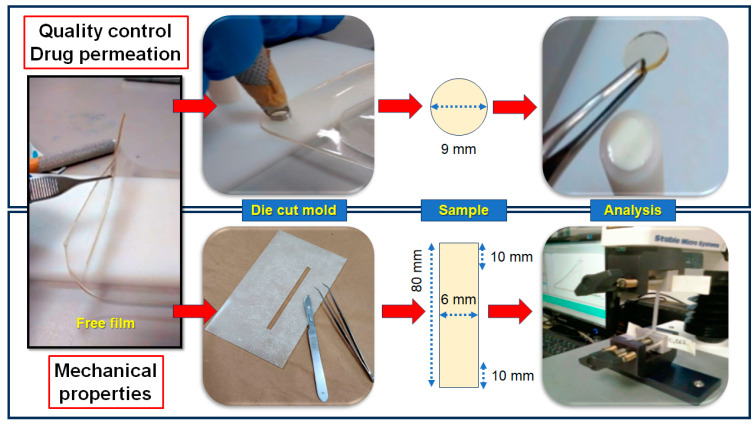
Illustration of the film sample preparation for the quality control assessment, in vitro permeation studies, and evaluation of mechanical properties.

The hydrogels were then poured into Petri plates (120 mm × 120 mm) and placed in a climatic chamber at 45 ± 5 °C and relative humidity of 50 ± 2% for 24 h to allow for solvent evaporation. After removal from the plates, the films were cut into circular specimens with a diameter of 9 mm using a stainless-steel punch (0.64 cm^2^, equivalent to 15 mg of the 1:1 drug mixture per unit) and stored in a desiccator with a relative humidity of approximately 50% obtained with a saturated solution of Mg(NO_3_)_2_·6H_2_O at room temperature until characterization. Temperature and humidity were monitored using a bench thermo/hygrometer. Figure 1 illustrates the sample preparation process. Throughout all experiments, investigators wore nitrile gloves to avoid film contamination with sweat or fat droplets from their hands.

The physicochemical quality attributes of the buccal anesthetic films, including uniformity of mass, thickness, and drug loading, were evaluated using the methods described previously [9]. All experiments were conducted at least in triplicate (*n* ≥ 3) to ensure reliability, and the results are presented as averages ± standard deviation (SD).

### 2.3. In Vitro Transmucosal Anesthetic Permeation

The use of porcine esophageal epithelium as a membrane barrier in buccal permeation experiments was first introduced by Del Consuelo et al. [64,65]. Our research team has validated this method for local anesthetics in a previous study [38].

The protocols for porcine esophageal epithelium excision, assessment of electrical resistance, mounting of diffusion cells, and HPLC-PDA simultaneous quantification of drug retention (Qepit, µg·cm^−2^) and permeation across the epithelium have been described in detail in our previous publications [9,37,38,54,55].

The release medium used was artificial saliva, composed of 2.38 g·L^−1^ Na_2_HPO_4_, 0.19 g·L^−1^ KH_2_PO_4_, and 8.0 g·L^−1^ NaCl, with a pH of 7.0. The artificial saliva was maintained under stirring conditions (300 rpm) within a temperature range of 25 ± 2 °C.

The composition of the artificial saliva was based on the previously published formulation by Mashru et al. [66], which is widely cited in release studies and recognized as a suitable simulated biological fluid for dissolution testing [67]. This composition was designed to create a buffer solution (pH 7.0) and maintain a favorable dissolution condition at a pH value compatible with healthy saliva [68].

**Figure 2:**
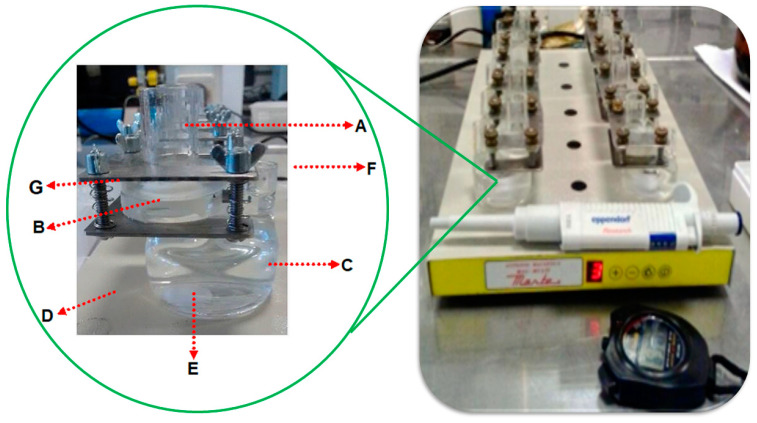
Vertical diffusion cells used in the transbuccal permeation experiments with prilocaine hydrochlorides and lidocaine hydrochloride from mucoadhesive films: (**A**) donor compartment; (**B**) porcine esophageal epithelium; (**C**) receiving compartment (35 mL); (**D**) magnetic stirrer; (**E**) magnetic bar; (**F**) sampling port; (**G**) sealing clip.

The release experiments (*n* = 5) were carried out using “Franz-type” vertical diffusion cells with a permeation area of 0.64 cm^2^ and a receptor compartment with an inner volume of 35 mL (Unividros, Ribeirão Preto, SP, Brazil). The parts of these diffusion cells and how they were assembled throughout the permeation experiments are displayed in Figure 2.

As recommended in previous studies [69], fresh porcine esophageal epithelium was used as the membrane barrier between the donor and receptor compartments. The epithelium was obtained from porcine esophagi collected immediately postmortem at a local slaughterhouse (Frigorífico Olhos D’água, Ipuã, SP, Brazil). Only epithelial tissues with electrical resistivity values of ≥3.0 KΩ·cm^2^ were selected for use in the experiments, as previously established [38,70]. This is to ensure the integrity and viability of the membrane barrier for accurate permeation studies.

Just before being placed into the cell donor compartments upon the membranes, the free surfaces of the release layers were hydrated with 50 µL of artificial saliva simulating its administration in clinical practice [8]. The samples (1.0 mL) of the dissolution medium were withdrawn every ten minutes for one hour, and the equivalent volume was immediately replenished. At each time point, the permeated amounts of the drugs were determined by HPLC-PDA [38]. The cumulative amounts of drug permeated were calculated according to Equation (1), as follows:

(1) $$Areal,t=\left(Cm,t\times Vr\right)\;+\left(\underset{n-1}{\sum }Cs\times Vs\right)$$

where *Areal*, *t* = the actual cumulative amount of drug permeated at time *t*; *Cm*, *t* = the determined concentration of the sample at time *t*; *Vr* = the volume of the receptor media; *Vs* = the volume of sample collected; and *Cs* = the concentration of sample collected.

Fick’s first law of diffusion was used to analyze the permeation kinetics of PCl and LCl. By plotting the cumulative amounts of permeated drugs (µg·cm^−2^) against time, we obtained a zero-order kinetics model that provided the following several key parameters: the steady-state permeation flux (*Jss*, µg·cm^−2^·min^−1^) was the slope of a linear regression model; the lag-time (*Lt*, min) was the ratio between the intercept of the linear regression model and *Jss*; and the permeability coefficient (*Kp*, cm·min^−1^) was the ratio between the *Jss* and the initial concentration of either PCl or LCl in the donor compartment (mg·g^−1^). Each experiment was performed in five independent replicates, and the results are presented as averages ± SD.

### 2.4. Assessing the Mechanical Properties

The mechanical properties of the film were evaluated using a tensile modulus TA XT plus texture analyzer (Stable Micro System, Surrey, UK) in agreement with a globally recognized method [71]. As summarized in Figure 1, a polycarbonate mold and stainless-steel blade were used to obtain film specimens (6 mm × 80 mm, width × length). It is noteworthy that each side of the specimens was cut with a single movement to avoid breaking points. Specimens presenting any fragile area were discarded.

Before analysis, the average thicknesses of every specimen were determined using a digital caliper by recording independent measures at six different points. The ends of the samples were positioned and fixed exactly in the center of the jaws in a vertical orientation. To prevent the grooves and creases in the equipment’s clamps from damaging its structure, the ends of the specimens (10 mm) were carefully covered with adhesive tape (Figure 1).

The following parameters were set: cell load was 50 kg (500 N), speed test was 1 mm·s^−1^, and maximum elongation was 190%. From the specimen dimensions (width [6 mm], actual length [60 mm], and average thickness) the Exponent Connect software generated stress vs. strain curves and determined the strength at break (FB, N), tensile strength (TS, MPa), elongation percentage at break (EB, %), and the Young’s modulus (YM, MPa) [72]. The analyses were performed at least in quintuplicate (*n* ≥ 5) at 25 ± 2 °C, and the results are presented as averages ± SD.

### 2.5. Data Analyses

The average data of three populations were compared by one-way analysis of variance (ANOVA) followed by Tukey’s post hoc test. The Student’s *t*-test was used to perform pairwise data comparisons. In all analyses, *p*-values of less than 5% (*p* < 0.05) at a 95% confidence interval were considered significant.

## 3. Results and Discussion

This study represents a proof of concept regarding the feasibility of α-bisabolol as a permeation enhancer of buccal local anesthetics from thin films. The research was motivated by the improved outcomes obtained with α-bisabolol in the dermal permeation of PHCl [57] and the retention of 5-ALA in the buccal epithelium [58]. Since both drugs were hydrophilic, such as by the aminoamide salts used, we hypothesized that dissolving this sesquiterpene in the film matrix and obtaining a viscous semisolid dispersion would favor drug interaction with the epithelium, enhancing drug retention by increasing the partitioning of drugs into the tissue [57], rather than impairing their dissolution.

During the preparation of the hydrogel, the addition of α-bisabolol in a dropwise manner resulted in a slightly whitish dispersion, resembling a nanoemulgel. Following solvent casting, the films exhibited homogeneity and lacked signs of drug crystallization, component segregation, fractures, or fragile areas. However, films F2 and F3 demonstrated increased adhesion to the plates and were slightly more challenging to handle due to their stickiness compared to F1. Moreover, F2 and F3 displayed a slightly more yellowish and brighter hue than F1, which may be attributed to the presence of α-bisabolol.

As presented in Table 2, the incorporation of α-bisabolol into the films did not significantly impact their physicochemical quality attributes, as no statistically significant differences were observed among them.

The PCl contents in F1, F2, and F3 averaged 90.6%, 101.1%, and 113.7%, respectively, of the expected contents (i.e., 7.5 mg of each drug at the 1:1 mixture), while the LCL content averaged 90.2%, 101.8%, and 113.2%, respectively. Therefore, a comprehensive risk matrix is essential for future scale-up studies to achieve 100% content uniformity. The thickness and drug content of the films varied within an acceptable range for buccal films (Table 2). Since the mass of the film sample can depend on the purpose of use and numerous formulation variables such as overall dimensions, drug loading, etc. Therefore, there is no general specification for this quality attribute.

**Figure 3:**
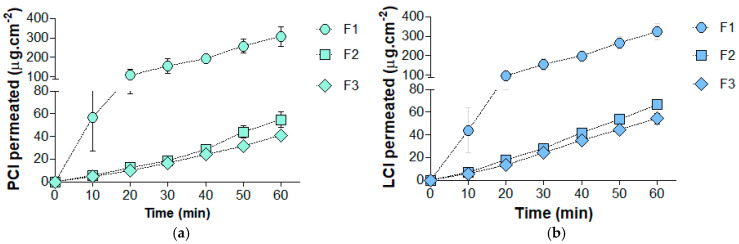
Cumulative permeation profiles of the local anesthetics (**a**) prilocaine hydrochloride (PCl) and (**b**) lidocaine hydrochloride (LCl) across porcine esophageal epithelium from the buccal films. F1—0% α-bisabolol; F2—15% α-bisabolol; F3—30% α-bisabolol; artificial saliva (35 mL); 300 rpm; and 25 ± 2 °C. Data expressed as averages ± SD (*n* = 5).

Figure 3a,b present the in vitro cumulative amounts of PCl and LCl, respectively, that permeated across the porcine esophageal epithelium from the buccal films. Additionally, Table 3 summarizes the permeation kinetics parameters determined for these local anesthetics. The permeation of both drugs fit very well with zero-order kinetics, as indicated by R² values > 0.99.

Unlike previous reports [58], α-bisabolol decreased the permeation and retention of both PCl and LCl through the porcine esophageal epithelium in this study. In summary, the addition of α-bisabolol reduced the *Jss* by approximately five-fold, the *Kp* by seven-fold, and the *Qepit* by two-fold for both PCl and LCl. Notably, no statistically significant differences were observed between the permeation parameters of F2 and F3 (*p* < 0.05), indicating that these effects were independent of the α-bisabolol concentration in the films.

The permeation of drugs through the oral mucosa’s epithelial tissue, which has a balance of polar and apolar lipids in its intercellular space, occurs primarily via the transcellular (intracellular) and paracellular (intercellular) pathways [83]. The paracellular pathway significantly impacts overall drug permeation through the oral epithelium [84], but the dominance of one pathway over the other depends on the drug’s physicochemical properties [83]. The paracellular pathway likely governs the permeation of PCl and LCl through the porcine esophageal epithelium, given their water-soluble biopharmaceutical properties [9]. Additionally, their slight differences in molecular mass, logP, and logD can also explain their distinct permeation kinetics. For clarification, the predicted physicochemical properties of PCl and LCl were calculated using Advanced Chemistry Development (ACD/Labs) software V11.02 (^©^1994–2012 ACD/Labs) for their base forms. 

In the absence of α-bisabolol (F1), the *Lt* for PCl was significantly lower than for LCl (*p* = 0.0111). This suggests that PCl, with its lower molecular mass (256.77 g·mol^−1^) and greater water solubility (logP of 2.029), diffuses faster from the polymeric matrix, is delivered more quickly to the surface, and crosses the epithelium faster than LCl (270.80 g·mol^−1^, logP of 2.196). These findings support the in silico method developed by Kokate et al. [84] for predicting drug permeability through the porcine buccal epithelium. According to this prediction tool, molecular volume is the primary descriptor affecting the permeability coefficient (logK) of drugs, including lidocaine, through the buccal mucosa, followed by lipophilicity (logD), number of hydrogen donor bonds, and number of rotational bonds.

According to in vitro/in vivo correlations reported by our research team [9], the penetration of PCl plays a crucial role in the onset of deep anesthesia and the maximum shallow anesthetic effect. Although the permeation and retention of both drugs through the epithelium were significantly impacted, at the higher concentration (F3), α-bisabolol significantly decreased *Jss* (*p* = 0.008) and increased *Lt* (*p* = 0.0146) of PCl compared to LCl. In turn, it is reasonable to consider that the drug partitioning in the epithelium was not differently affected by α-bisabolol, as the cumulative amounts of drug permeated (Qepit) of PCl and LCl did not differ significantly in F2 and F3. Overall, PCl was shown to be more negatively affected than LCl, which may reduce the efficacy of the films in clinical practice.

At the studied concentrations, because of its remarkable lipophilicity (logP 5.1) [85], α-bisabolol may have decreased the film’s hydrophilicity and hydration, thereby lessening drug dissolution, thermodynamic activity (concentration) at the mucosal surface, diffusion, and, finally, permeation across the epithelium through the paracellular pathway. Since the actual hydrophilicity of the developing films remains unexplored, further experiments are warranted to confirm or refute this hypothesis.

One limitation of this study is that we did not quantify the release of α-bisabolol from the films. However, high-resolution HPLC-PDA analysis [38] revealed no additional peak compounds in the films containing α-bisabolol compared to those without it (F1), suggesting that α-bisabolol did not permeate through the porcine esophageal epithelium and lost its enhancement capacity. Additionally, Table 2 shows no significant statistical difference in thickness among the various groups analyzed. Therefore, we conclude that thickness does not impact drug release and permeation, and the varying content of α-bisabolol is the sole contributing factor.

To avoid pH changes throughout the experiments, the release medium (artificial saliva) was balanced with Na_2_HPO_4_, KH_2_HPO_4_, and NaCl, and the resulting buffer pH was adjusted to 7.0. Assuming the drugs are weak organic bases (pKa of 7.89 and 7.86 for prilocaine and lidocaine, respectively), since the drug loading in the films was low (low mol number), even achieving total dissolution in the release medium (35 mL, resulting in 429 µg·mL^−1^), it would not be able to change the pH significantly. Moreover, the release medium replacement following sampling reinforces the hypothesis of maintaining a constant pH; therefore, this factor did not affect drug permeation.

Patches containing a drug delivery layer with a composition similar to F1 but with a higher drug loading (30 mg of PCl and LCl, 1:1) have been tested on adult volunteers who underwent routine medium-complexity dental procedures [8]. The efficacy was 90%, and in successful cases, there was no need for complementary conventional infiltrative local anesthesia during the procedures. Notably, patients did not report any discomfort or side effects during or after the intervention, while decreasing their anxiety. Such patches presented a fast onset (5 min) in most cases. The maximum anesthetic effect was reached within 15 and 25 min, and the anesthetic effect lasted at least 50 min, making it suitable for clinical practice.

The reduced final volume and optimized shape of the developing F1 can enable multiple administrations simultaneously at selected regions. As a result, the amount of drug available for permeating the buccal tissue can be tuned. Therefore, even with a longer delivery rate than infiltrative anesthesia, these films may provide effective, comfortable, painless, and safe outcomes in routine dental procedures.

Regarding the mechanical properties, the addition of α-bisabolol made the films less resistant and flexible (Table 2). These effects did not depend on the concentration of this phytochemical, as there was no statistically significant difference between the results obtained for F2 and F3 (*p* < 0.05). On average, α-bisabolol decreased the EB, TS, and FB by four-fold, three-fold, and two-fold, respectively. Furthermore, the increase in the YM provided by adding α-bisabolol was 38% on average.

EB measures the extent to which a film can stretch before breaking, indicating the flexibility of the film. A higher EB suggests that the film can stretch significantly more before breaking, which is important for films that need to conform to the contours of the buccal cavity without tearing. TS reflects the overall strength of the film, as it is the maximum stress that the film can withstand while stretching before breaking. A higher TS indicates a stronger film that can resist breaking under tension, crucial for ensuring the film remains intact during handling and application. FB is the force required to break the film, providing a direct measurement of the resistance to breaking. YM measures the stiffness of films; a higher Young’s modulus indicates a stiffer and less flexible film, while a low modulus indicates a more flexible film [80].

Table 2 reveals that there are scant literature reports on the mechanical properties of buccal anesthetic films, which can vary widely depending on factors such as drug loading, type and loading of plasticizer, type and amount of film-forming polymer, overall sample dimensions, and experimental setup [72,82,86,87].

Balancing flexibility and strength is crucial for needle-free buccal anesthetic films. Ideally, these films should be sufficiently strong to handle and apply without tearing but flexible enough to adapt to the movements and shape of the buccal cavity, ensuring comfort and effective drug delivery [8,9]. Among the most common behaviors of films as assessed by stress–strain curves [88], i.e., (i) hard and brittle (high YM [>1000 MPa], moderate to high TS [20 to 100 MPa], and FB [20–100 N] and low EB [<50%]); (ii) hard and strong (high YM [>1000 MPa], TS [>100 MPa], and FB [>100 N] and low to moderate EB [50 to 200%]); (iii) soft and weak (low TS [<20 MPa], YM [<100 MPa], and FB [<10 N] and high EB [>200%]); and (iv) soft and tough (moderate to high TS [10 to 100 MPa], low to moderate YM [100 to 500 MPa], high EB [>200%], and moderate to high FB [20 to 100 N]); the latter better fits the ideal properties of buccal anesthetic films. While F1 presented moderate EB, TS, and FB and low YM (soft and tough), F2 and F3 presented low EB, TS, FB, and YM (soft and weak).

In conclusion, the addition of α-bisabolol at concentrations of 0.9% and 1.8% (equivalent to 15% and 30% of the drug content, respectively) did not enhance the permeation or retention of prilocaine and lidocaine hydrochlorides across the porcine esophageal epithelium. Instead, it compromised the mechanical properties of the drug-loaded HPMC™ K100 films. Our findings suggest that buccal films containing α-bisabolol may not be suitable for use as needle-free local anesthetic delivery systems in routine dental procedures. Although our results contradict the initial hypothesis, they confirm that the permeation of hydrophilic drugs across the porcine esophageal epithelium can be affected differently by varying concentrations of this sesquiterpene.

## Figures and Tables

**Table 1:** Composition of the buccal anesthetic films.

Component (%, m·m^−1^)	Films		
	F1	F2	F3
PCl	3.0	3.0	3.0
LCl	3.0	3.0	3.0
HPMC K100 LV	3.0	3.0	3.0
PEG 400 *	0.9	0.9	0.9
α-Bisabolol **	-	0.9	1.8
PBS pH 7.0	qsp 100	qsp 100	qsp 100

F1—0% α-bisabolol; F2—15% α-bisabolol; F3—30% α-bisabolol; HPMC—hydroxypropyl methylcellulose; PEG—polyethylene glycol; PCl—prilocaine hydrochloride; LCl—lidocaine hydrochloride; qsp—quantity sufficient for preparation. * Equivalent to 15% (m·m^−1^) of the total drug content; ** equivalent to 0, 15, or 30% of the total drug content (m·m^−1^).

**Table 2:** Physicochemical quality attributes and mechanical properties of the buccal anesthetic films.

		Films			
		F1	F2	F3	Literature Specification
Quality attributes	Mass (mg)	18.9 ± 2.6	17.7 ± 0.4	18.9 ± 0.5	Changeable [9,45,48,55,73,74,75,76,77,78,79]
	Thickness (µm)	292.5 ± 61.0	344.8 ± 34.0	397.6 ± 49.0	50 to 1000 µm ^$^ [80]
	PCl content (mg·g^−1^)	291.7 ± 21.0	344.6 ± 22.0	381.6 ± 6.3	85–115% ^$^ [81]
	LCl content (mg·g^−1^)	291.2 ± 15.1	347.4 ± 24.0	379.5 ± 5.0	85–115% ^$^ [81]
Mechanical properties	EB (%)	123.0 ± 4.0	31.6 ± 3.6 ^C^	33.5 ± 5.2 ^C^	0.9–137.1 [72,82]
	TS (MPa)	10.0 ± 1.0	3.5 ± 0.3 ^C^	3.5 ± 0.8 ^C^	0.6–70.4 [72,82]
	FB (N)	18.0 ± 4.0	7.6 ± 0.7 ^C^	8.2 ± 2.3 ^C^	2.3–34.8 [72,82]
	YM (MPa)	0.08 ± 0.005	0.11 ± 0.004 ^A^	0.11 ± 0.02 ^A^	−14.4–9.7 [72]

F1—0% α-bisabolol; F2—15% α-bisabolol; F3—30% α-bisabolol; PCl—prilocaine hydrochloride; LCl—lidocaine hydrochloride; EB—elongation at break; TS—tensile strength; FB—force at break; Ym—Young’s Modulus. ^$^ General specification for buccal films. The results are expressed as averages ± SD (*n* ≥ 3). Significant statistical differences at ^A^ (*p* < 0.05) and ^C^ (*p* < 0.001) compared to the correspondent value of F1 (at a confidence interval of 95%) as determined by one-way ANOVA followed by Tukey’s post hoc test.

**Table 3:** Kinetics permeation parameters and amount of local anesthetic retained in the porcine esophageal epithelium from the buccal anesthetic films.

	Films		
Parameter	F1	F2	F3
*J_SS_* PCL (µg·cm^−2^·min^−1^)	5.0 ± 0.3	1.0 ± 0.2 ^C^	0.7 ± 0.1 ^C^
*J_SS_* LCL (µg·cm^−2^·min^−1^)	5.6 ± 0.4	1.2 ± 0.1 ^C^	1.0 ± 0.1 ^C^
*Lt* PCl (min)	1.7 ± 0.3	7.2 ± 3.4 ^A^	5.8 ± 0.2 ^A^
*Lt* LCl (min)	4.0 ± 1.0	4.5 ± 1.1	5.1 ± 0.1
*Kp* × 10^−4^ PCL (cm·min^−1^)	8.9 ± 0.8	1.4 ± 0.3 ^C^	0.9 ± 0.1 ^C^
*Kp* × 10^−4^ LCL (cm·min^−1^)	9.9 ± 0.7	1.7 ± 0.3 ^C^	1.2 ± 0.2 ^C^
*Qepit* PCL (µg·cm^−2^)	1277.8 ± 53.4	567.9 ± 55.9 ^C^	522.8 ± 69.4 ^C^
*Qepit* LCL (µg·cm^−2^)	1254.4 ± 34.1	696.8 ± 83.3 ^C^	625.1 ± 97.9 ^C^

F1—0% α-bisabolol; F2—15% α-bisabolol; F3—30% α-bisabolol; PCl—prilocaine hydrochloride; LCl—lidocaine hydrochloride; *Jss*—steady-state permeation flux; *Lt*—lag time; *Kp*—permeability coefficient; *Qepit*—amount of drug retained in the porcine esophageal epithelium. Data are expressed as averages ± SD (*n* = 5). Significant statistical differences at ^A^ (*p* < 0.05) and ^C^ (*p* < 0.001) compared to the correspondent value of F1 (at a confidence interval of 95%) as determined by one-way ANOVA followed by Tukey’s post hoc test.

## Data Availability

The raw data supporting the conclusions of this article will be made available by the authors upon request.
